# Cathelicidin-derived antiviral peptide inhibits herpes simplex virus 1 infection

**DOI:** 10.3389/fmicb.2023.1201505

**Published:** 2023-06-05

**Authors:** Xiaomin Guo, Yanxing An, Wanmin Tan, Ling Ma, Mingyang Wang, Juyan Li, Binghong Li, Wei Hou, Li Wu

**Affiliations:** ^1^College of Veterinary Medicine, Shanxi Agricultural University, Jinzhong, China; ^2^Department of Medical Imaging, First Affiliated Hospital of Kunming Medical University, Kunming, China

**Keywords:** cathelicidins, antiviral peptide, HSV-1, facial palsy, WL-1

## Abstract

Herpes simplex virus 1 (HSV-1) is a widely distributed virus. HSV-1 is a growing public health concern due to the emergence of drug-resistant strains and the current lack of a clinically specific drug for treatment. In recent years, increasing attention has been paid to the development of peptide antivirals. Natural host-defense peptides which have uniquely evolved to protect the host have been reported to have antiviral properties. Cathelicidins are a family of multi-functional antimicrobial peptides found in almost all vertebrate species and play a vital role in the immune system. In this study, we demonstrated the anti-HSV-1 effect of an antiviral peptide named WL-1 derived from human cathelicidin. We found that WL-1 inhibited HSV-1 infection in epithelial and neuronal cells. Furthermore, the administration of WL-1 improved the survival rate and reduced viral load and inflammation during HSV-1 infection via ocular scarification. Moreover, facial nerve dysfunction, involving the abnormal blink reflex, nose position, and vibrissae movement, and pathological injury were prevented when HSV-1 ear inoculation-infected mice were treated with WL-1. Together, our findings demonstrate that WL-1 may be a potential novel antiviral agent against HSV-1 infection-induced facial palsy.

## 1. Introduction

Herpes simplex virus 1 (HSV-1), a member of the family Herpesviridae, subfamily Alphaherpesvirinae, is an enveloped virus with a double-stranded (ds) DNA genome consisting of a non-spherical capsid, cortex, and capsule (Whitley and Roizman, 2001; Rechenchoski et al., 2017). HSV-1 is a ubiquitous but important human pathogen. Recent epidemiologic studies estimate that over half of the world's population is infected with HSV-1, making it a global health concern (James et al., 2020; Imafuku, 2023). HSV-1 initially prefers to infect the genital and oral mucosa. Painful blisters or ulcers at the site of infection are caused by contagious and long-lasting infections. The virus can then migrate to the sensory ganglia and enter the latent stage, preventing clearance by the immune system (McQuillan et al., 2018). In brief, HSV-1 infection begins with primary infection in the periphery, followed by lifelong latency in the peripheral nervous system, which can cause various clinical signs and symptoms, such as skin lesions, acute retinal necrosis, genital sores, and other pathologies (Khalesi et al., 2023). Furthermore, HSV-1 can cause fatal systemic infections or encephalitis, problems typically most associated with immune naive or immunocompromised patients (Imafuku, 2023). HSV-1 has been reported to be the most common cause of infectious blindness and fatal encephalitis worldwide. It can also cause Bell's palsy when it infects the facial nerve, an acute spontaneous facial paralysis that accounts for 50–70% of all peripheral facial paralysis (Imafuku, 2023; Khalesi et al., 2023). Reactivation of latent HSV-1 infection in the geniculate ganglion is considered a major cause of Bell's palsy. Approximately 30% of patients with Bell's palsy are still at risk for continued facial paralysis and pain, while most people can recover fully on their own (Zhang et al., 2023). The majority of treatment drugs for HSV-1 are acyclovir, a nucleoside analog, and its derivatives, as these compounds are activated by the viral thymidine kinase (TK) and inhibit the viral polymerase, resulting in preventing the production of infectious virions (Aribi Al-Zoobaee et al., 2020; Stoyanova et al., 2023). The latent stage and the development of resistance are limitations to the use of these drugs. However, ACV can be responsible for renal cytotoxicity with acute renal failure requiring dialysis (Neto et al., 2007). Additionally, the drug-resistant HSV-1 strains, especially acyclovir-resistant strains, which are related to mutations of the viral TK or DNA polymerase genes, have been the major challenges facing HSV-1 treatment (Gu et al., 2014). Consequently, new cure strategies are needed, and to do so, it is necessary to develop new effective anti-HSV-1 drugs.

Antimicrobial peptides have received increasing attention from the scientific community over the last 20 years due to the worldwide increase in antibiotic resistance among microorganisms. At present, more than 3,200 natural antimicrobial peptides have been reported in the antimicrobial peptide database (https://aps.unmc.edu). Antimicrobial peptides (AMPs) are molecules present in the innate immune system of almost all living organisms, invertebrates, and vertebrates and have been identified as potential agents with therapeutic potential as they exhibit marked antibacterial, antiviral, antiparasitic, and antifungal properties (Zasloff, 2002; Barashkova and Rogozhin, 2020; Memariani et al., 2020; De Angelis et al., 2021). Cathelicidin is a member of the AMPs family, which is a part of the immune system and can be produced by a variety of eukaryotic organisms (Chessa et al., 2020). They are well-conserved during genome evolution and have similar modes of action (Zhang et al., 2023). Cathelicidins have revealed potent antimicrobial activity against bacteria and viruses, which has gradually become an interesting and promising research topic (Cebrián et al., 2023). Cathelicidins are characterized by two functional domains, namely the conserved cathelin-like proregion and the N-terminal active domain region (Kościuczuk et al., 2012). Several studies have demonstrated that cathelicidins exhibit potent anti-endotoxin properties *in vitro* and *in vivo*, both by binding bacterial LPS and by intervening in TLR signaling mechanisms (Rosenfeld et al., 2006). Their antiviral mechanisms include the inhibition of viral entry, intracellular viral replication, and assembly and induction of the immune response. For instance, CRAMP, a cathelicidin identified in mouse, can affect the survival and replication of the influenza A virus (IAV) in host cells by directly disrupting the IAV envelope (Gallo et al., 1997). PG-1, an antimicrobial peptide of the cathelicidins family, has been reported to inhibit viral infection by blocking the adsorption of porcine reproductive and respiratory syndrome virus (PRRSV) on embryonic kidney cells of African green monkey (Guo et al., 2015). In addition, they show a lower tendency to induce resistance than conventional bacterial antibiotics (Mehmood Khan et al., 2023). As a consequence, they are a new generation of antiviral biomolecules with very low toxicity to human host cells and play a role in the treatment of a variety of diseases and symptoms, which can be considered an appropriate choice for the treatment of resistant pathogens in the future (Chen et al., 2013; AlMukdad et al., 2023; Baindara et al., 2023).

LL-37, a peptide derived from the human cathelicidin, has been shown to resist HSV-1 infection (Lee et al., 2014; Roy et al., 2019), whereas LL-37 is limited in its therapeutic use due to its length of 37 residues, resulting in high chemical synthesis costs. Herein, we designed a 16-amino acid peptide, WL-1, which is based on human cathelicidin LL-37 and its fragments (Li et al., 2006; He et al., 2018). Moreover, we displayed the antiviral activity of WL-1 against HSV-1 infection. Furthermore, the administration of WL-1 ameliorated the pathological symptoms of inflammation and facial paralysis induced by HSV-1 infection. In summary, this study shows that WL-1 may be a potent agent candidate against HSV-1 infection.

## 2. Materials and methods

### 2.1. Mice

C57 female mice (8-week-old, weighing 17–19 g) were purchased from SPF (Beijing, China) Biotechnology Co., Ltd. Mice were group-housed at room temperature and a 12-h light/dark cycle, with free access to water and standard animal food. The animal care and experimental protocol were approved by the Animal Care Committee of Shanxi Agricultural University, approval number: SXAU-EAW-2022M.RX.00906001.

### 2.2. Cells, viruses, and peptides

U251 cells and Vero cells were obtained from the Kunming Cell Bank, Kunming Institute of Zoology, Chinese Academy of Sciences. All cells were cultured in a DMEM medium (Gibco, Waltham, MA, USA) supplemented with 10% fetal bovine serum (FBS), 100 U/mL of penicillin, and 100 μg/ml of streptomycin in 5% CO_2_ at 37°C. HSV-1 was stored at −80°C in our laboratory. The peptide WL-1 (GWKRIKQRIKDKLRNL) was designed based on human cathelicidin LL-37 and the existing derived GF-17 (He et al., 2018). Three residues were redesigned (F2W, V6K, and F12K), and one residue was removed (V17) in combination with C-terminal amidation (-NH2) compared to GF-17. It was synthesized by GL Biochem (Shanghai) Ltd. (Shanghai, China). The peptide was analyzed by reversed-phase high-performance liquid chromatography (RP-HPLC) and mass spectrometry. The purity of WL-1 was over 98%.

### 2.3. Cytotoxicity assay

The cytotoxicity of WL-1 was tested on U251 and Vero cells. The cells were seeded in a 96-well plate with 2 × 10^5^ cells/ml for 12 h, and a certain amount of WL-1 was added (the final concentration increased gradually in the range of 0–250 μM). After incubation for 24 h, cell viability was evaluated by conventional 3-(4,5-dimethyl-2-thiazolyl)-2, 5-diphenyl-2H-tetrazolium bromide (MTT) reduction assays. In detail, 20 μl of MTT (5 mg/mL) was added to each well. The MTT solution was then removed, and 200 μl dimethyl sulfoxide (DMSO) was added to solubilize the MTT-formazan crystals in living cells. The absorbance at 570 nm of the resulting solution was measured.

### 2.4. Plaque-forming assays

HSV-1 (MOI = 0.1) was first co-cultured with different concentrations of WL-1 (0, 2, 10, and 50 μM) or acyclovir (50 μM) for 2 h and then diluted to infect Vero cells. The maintenance medium containing 1% methylcellulose was used to replace the cell culture medium. After 3 days of incubation, 10% formalin was used to fix the cells, and 1% crystal violet was subsequently used to stain the cells. Finally, the plaque-forming units were counted.

### 2.5. Cell infection

Vero and U251 cells were seeded in a 12-well plate with 5 × 10^5^ cells/well for 12 h and then infected with HSV-1 at 0.1 MOI combined with WL-1 (0, 2, 10, 50 μM) or acyclovir (50 μM) administration for 24 h. Cells were then lysed in a TRIzol reagent (TIANGEN, Beijing, China), and the total RNA was extracted under the manufacturer's instructions. The relative expression levels of HSV-1 (HSV-1, UL27, UL52, and UL54) were detected by RT-qPCR using GAPDH as a reference gene.

### 2.6. Quantitative real-time PCR (RT-qPCR)

For the RT-qPCR analysis, total RNA was isolated from cells, and cDNA was reverse-transcribed by using M-MLV reverse transcriptase (Promega, Madison, WI, USA). RT-qPCR was performed on the StepOnePlus Real-Time PCR Systems (Thermo, Waltham, MA, USA). The primers used are shown in Table 1 (Liu et al., 2023). Primer was designed to target the ICP0 gene of HSV-1 to represent virus replication.

**Table 1:** Sequences of primers used for RT-qPCR.

**Gene**	**Forward (5^′^-3^′^)**	**Reverse (5^′^-3^′^)**
HSV-1 (ICP0)	CCCACTATCAGGTACACCAGC	CTGCGCTGCGACACCTTTT
UL27	GCCTTCTTCGCCTTTCGC	CGCTCGTGCCCTTCTTCTT
UL52	AGGCCATCAAGGACATCTGC	AATACGGCGCTCCACGTAAA
UL54	TGGCGGACATTAAGGACATTG	TGGCCGTCAACTCGCAGA
GAPDH	CACCATCTTCCAGGAGCGAG	AGAGGGGGCAGAGATGATGA

### 2.7. Mice infection

For ocular scarification infection reported in other studies (Li et al., 2016), mice were divided into four groups: HSV-1 only as the model group, HSV-1 and WL-1 (5 mg/kg) as the experimental group, HSV-1 and acyclovir (5 mg/kg) as the positive control group, and PBS as the negative control group. The infection dose of HSV-1 was 4 × 10^4^ plaque forming units (PFU). WL-1 was treated via tail vein injection after 1 h of HSV-1 infection. Mice were weighed and monitored daily over time. The survival rate of mice was observed by day 20. Mice were euthanized on day 5 after infection, and brains were harvested to determine the viral burden by plaque assay. In addition, enzyme-linked immunosorbent assay (ELISA) was used to detect the content of inflammatory factors in serum and brain.

Moreover, HSV-1 infection via ear inoculation was used to induce facial paralysis (Takahashi et al., 2001). Following anesthesia with an intraperitoneal injection of sodium pentobarbital (50 mg/kg), the posterior surface of the left auricle was scratched 20 times with a 27-gauge needle, and then, 25 μl of virus solution (1.0 × 10^6^ PFU) was placed on the scratched area. The negative control group was injected with normal saline, and the positive control group and the experimental group were injected with HSV-1. Then, the experimental group was treated with WL-1 (5 mg/kg) via tail vein injection after 1 h of HSV-1 infection. Next, blink reflex, vibrissae movements, and nose tip position were carefully observed every 12 h so as to evaluate the facial paralysis. The blink reflex was performed by blowing air into the eye at a distance of 2 cm away from the eye by the use of a 5 ml syringe without a needle, and the movement and closure condition of the eyelid were observed. The time of vibrissa movement of the inoculated side in 30 s was counted, compared with that of the contralateral side. Meanwhile, the nose tip position was recorded. After 72 h, tissue from the facial nerve and its connection to the brain was cut and observed. We also performed an inflammatory score.

### 2.8. ELISA

The brains of infected mice as described above were excised and then homogenized in 0.9% NaCl with a glass homogenizer. The supernatant was collected after centrifugation at 2,000 *g* for 15 min. The serum was obtained by centrifugation of blood at 3,000 rpm for 10 min. The concentration of TNF-α and IL-6 in the supernatant and serum harvested above was measured by using enzyme-linked immunosorbent assay kits (Dakewe, Beijing, China) according to the manufacturer's instructions.

### 2.9. Statistical analysis

Data are given as mean ± SEM. Statistical analysis was performed using a two-tailed Student's *t*-test and log-rank test. A *p*-value of < 0.05 was considered to be statistically significant.

## 3. Results

### 3.1. Cytotoxicity of WL-1

**Figure 1:**
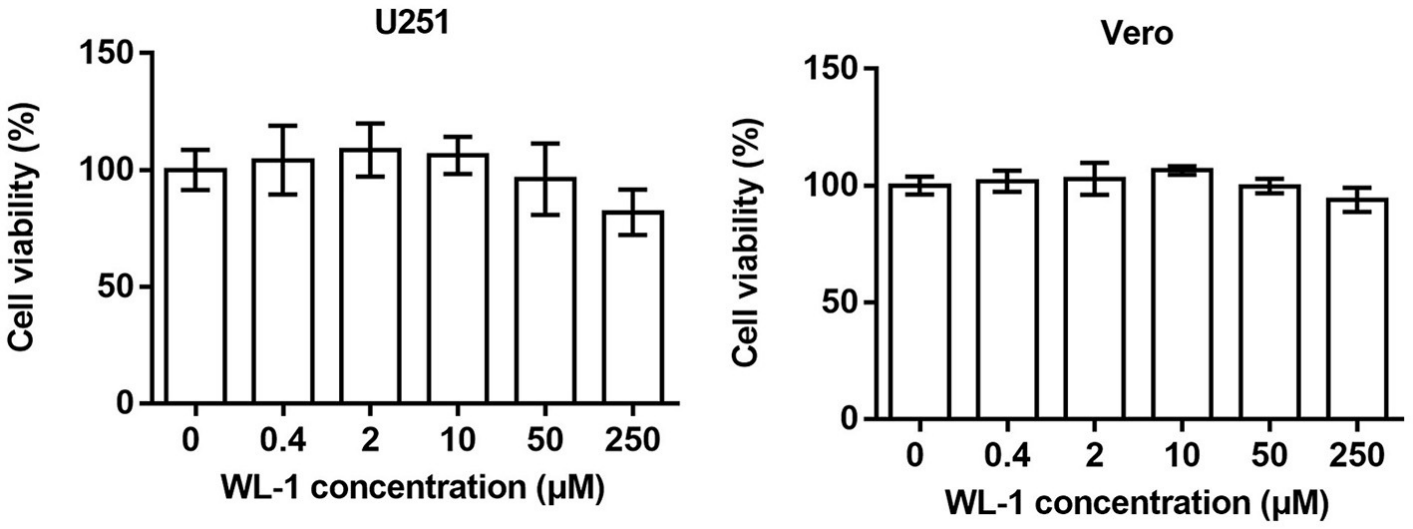
Cytotoxicity of WL-1. U251 and Vero cells were treated with increasing concentrations of WL-1 (0, 0.4, 2, 10, 50, and 250 μM), and then, cell viability was detected after 24 h culture by using MTT assays. Data represent three independent experiments and are presented as mean ± SEM.

To distinguish antiviral activity from cellular toxicity, the cytotoxicity of WL-1 was tested on various HSV-1 host cells, including neuronal cell (U251) and epithelial cell (Vero; Figure 1). Cells were treated with different concentrations of WL-1 (0, 0.4, 2, 10, 50, and 250 μM) for 24 h, and the cell viability was analyzed by MTT assays. We found that the cell viability was reduced by no more than 10% by 50 μM WL-1 in all cell lines, which suggested that WL-1 did not exert toxic effects on cells up to 50 μM concentration. Therefore, a concentration of WL-1 below 50 μM was used for the subsequent research.

### 3.2. WL-1 inhibits HSV-1 infection *in vitro*

**Figure 2:**
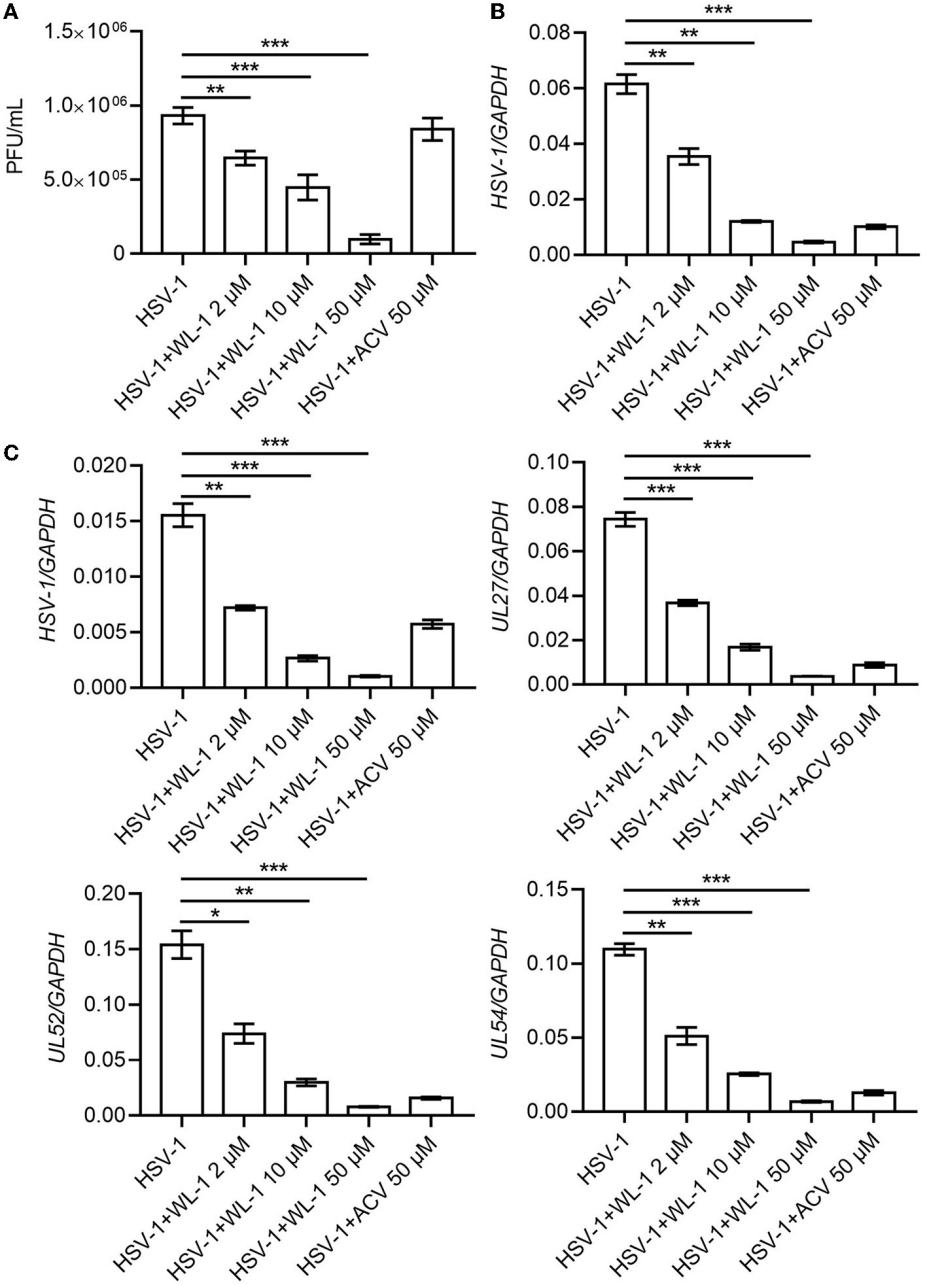
WL-1 inhibits HSV-1 in cells. **(A)** The HSV-1 (MOI = 0.1) virus was co-cultured with different concentrations of WL-1 or acyclovir (labeled as ACV) for 2 h, and then, plaque-forming units were detected. **(B)** Vero cells were infected with HSV-1 (MOI = 0.1) with different concentrations of WL-1 or ACV for 24 h, and the viral gene was analyzed by RT-qPCR. **(C)** U251 cells were infected with HSV-1 (MOI = 0.1) with different concentrations of WL-1 (0, 2, 10, and 50 μM) or acyclovir (labeled as ACV) for 24 h, and the viral genes were analyzed by RT-qPCR. Data represent three independent experiments and are presented as mean ± SEM. *p < 0.05; **p < 0.01; ***p < 0.001.

To determine whether WL-1 has direct anti-HSV-1 activity, HSV-1 was incubated with different concentrations of WL-1, and plaque assay was performed on Vero cells. As shown in Figure 2A, the viral plaque number was significantly decreased by WL-1 in a dose-dependent manner. Only half of the infectious particles were present in the cells treated with WL-1 at 10 μM concentration with a 50% antiviral activity at this dose, which suggested that WL-1 reduces the viable viral load. In order to further assess the antiviral activity of WL-1, epithelial cells Vero were infected with HSV-1 at 0.1 MOI for 24 h in the presence or absence of WL-1. Acyclovir, a major clinical drug used in the treatment of HSV-1 infection, was chosen as a positive control (Gurgel Assis et al., 2021). Notably, administration of WL-1 decreased the intracellular load of HSV-1 in Vero cells (Figure 2B). To confirm these results, we also examined the antiviral effects of WL-1 on neuronal U251 cells, an HSV-1-sensitive cell line. Similarly, WL-1 significantly inhibited the replication of HSV-1. In addition, RT-qPCR analysis showed that WL-1 markedly reduced the expression of viral late gene UL27, early gene UL52, and immediate early gene UL54. In addition, the effect of HSV-1 inhibition became stronger with increasing WL-1 concentration (Figure 2C). The antiviral activity of WL-1 was equivalent to or even better than that of acyclovir at an equal concentration on all experiment cells. Taken together, these results indicate the strong antiviral effect of WL-1 against HSV-1 infection, which depends on the dose of WL-1 within a certain concentration range.

### 3.3. WL-1 suppresses HSV-1 infectivity in mice

**Figure 3:**
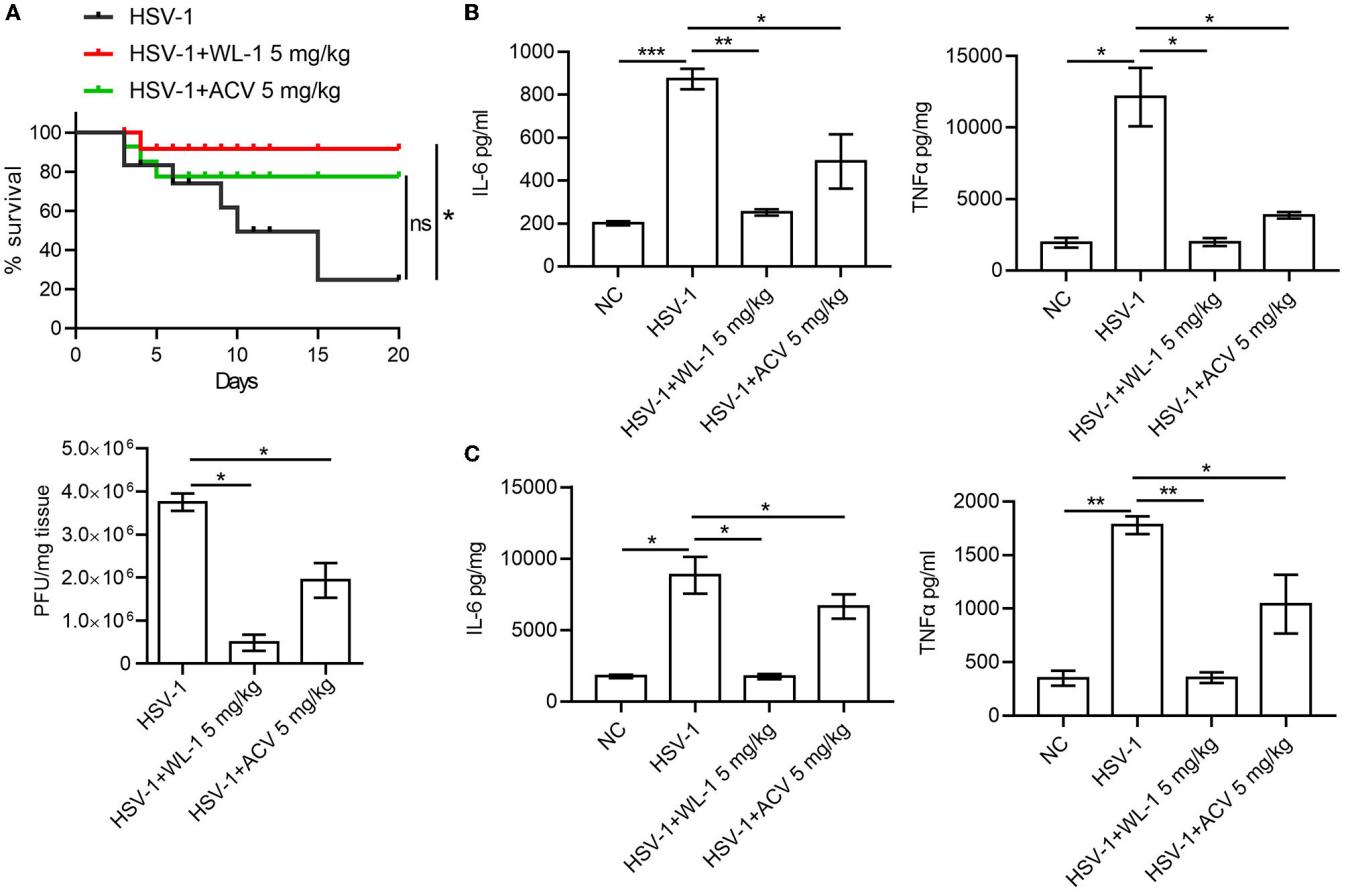
WL-1 inhibits HSV-1 infection *in vivo*. **(A)** Mice were infected via ocular scarification with 4 × 10^4^ PFU HSV-1 and then administered with WL-1 (5 mg/kg) or acyclovir (5 mg/kg; labeled as ACV) 1 h after infection. Survival rates were checked daily until day 20, and brain virus loads were measured by plaque assay 5 days after infection. **(B, C)** The production of IL-6 and TNF-α in serum **(B)** and the brain **(C)** 5 days postinfection of HSV-1 was measured by ELISA. Data represent two independent experiments and are presented as mean ± SEM. *p < 0.05; **p < 0.01; ***p < 0.001; ns, not significant.

To define the role of WL-1 against HSV-1 infection *in vivo*, wild-type mice were infected with 4 × 10^4^ PFU of HSV-1 to each eye (via ocular scarification) with or without WL-1 administration, and survival was monitored over time. Mortality of normal infected mice occurred at 3 days postinfection, while infected mice with WL-1 administration began to die at 4 days postinfection. In addition, the survival rate of HSV-1-infected mice in the presence of WL-1 was ~90% by day 20, which was markedly higher than that of mice without WL-1 administration (Figure 3A). To investigate whether the attenuated pathogenesis noted above was due to a decreased burden of HSV-1 in the mouse with WL-1 administration, we analyzed viral loads of HSV-1 in the brain after 2 days of infection. Plaque assay revealed that a lower level of virus titer was observed in the brain of HSV-1-infected mice following the administration of WL-1 than those mice in the absence of WL-1 (Figure 3A). Moreover, we detected the content of inflammatory cytokines in the peripheral blood and brain, which is a hallmark of the immune response to pathogenic infection. Strikingly, the production of inflammatory cytokines IL-6 and TNF-α in mice induced by HSV-1 infection was substantially reduced in the presence of WL-1 or acyclovir in the peripheral blood (Figure 3B). In line with these findings, the production of IL-6 and TNF-α in the brain was significantly decreased by the administration of WL-1 or acyclovir during infection with HSV-1 (Figure 3C). Overall, HSV-1 infection in the presence of WL-1 administration resulted in increased survival and decreased replication and inflammation in the host, suggesting that the WL-1 has the ability to suppress HSV-1 infection.

### 3.4. WL-1 prevents facial palsy induced by HSV-1

**Figure 4:**
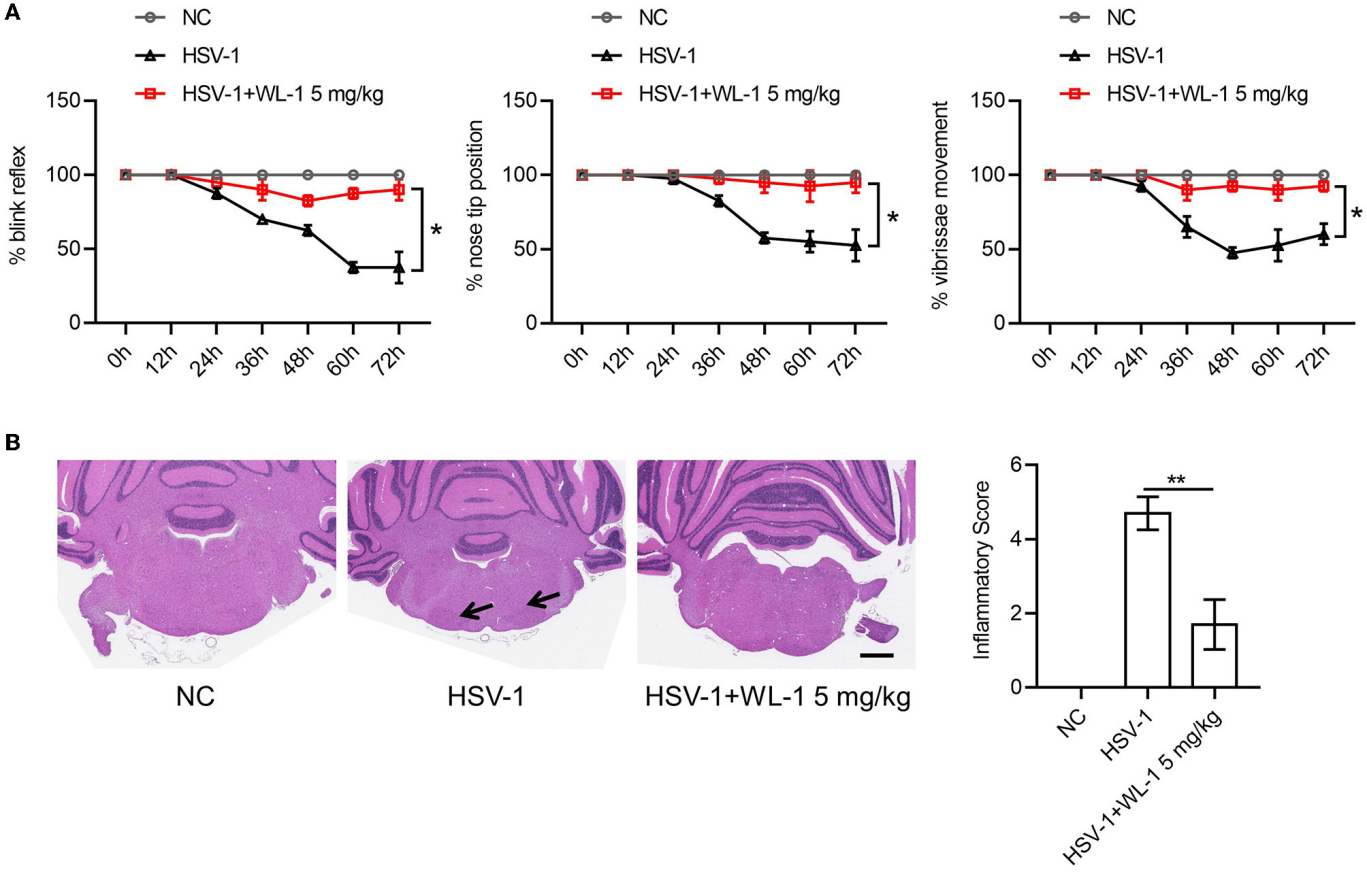
WL-1 protects mice from facial palsy induced by HSV-1. **(A)** Mice were infected *via* ear inoculation to induce facial paralysis with 25 μl of virus solution (1.0 × 10^6^ PFU) and then administered with WL-1 (5 mg/kg) or PBS 1 h after infection. Blink reflex, nose tip, and vibrissae movement were observed every 12 h postinfection. **(B)** Representative facial nerve pathological section from mice in **(A)**. The arrow indicates inflammatory cell infiltration. Data represent two independent experiments and are presented as mean ± SEM. Scale bars, 1,000 μm. *p < 0.05; **p < 0.01.

Published studies report that HSV-1 can infect the host in various manners to cause different symptoms (Honda et al., 2002; Kastrukoff et al., 2012; Lee et al., 2015; Caliento et al., 2018). An animal disease model of HSV-1 infection has been developed to study Bell's palsy caused by HSV-1 reactivation. To examine whether the protective role of WL-1 on HSV-1 was specific to the infection method and whether WL-1 also had an effect on facial palsy, HSV-1 was infected via ear inoculation known to induce facial palsy with the administration of WL-1 and facial nerve function indicators were evaluated up to day 3. As reported, HSV-1 infection resulted in a series of facial nerve dysfunctions, including loss of blink reflex, unnatural nose position, and weakness of vibrissae movement. There was no significant difference in the time to onset of abnormal blink reflex between the WL-1 administration and the control group. Instead, the ratio of the normal blink reflex in WL-1 treated mice was significantly higher than that in untreated mice. The nose tip position and vibrissae movement of HSV-1 alone infected mice were abnormal from the 36th h postinfection, but almost all the WL-1 treated mice were normal throughout the infection period. In addition, the administration of WL-1 also markedly increased the percentage of mice with normal nose position and vibrissae movement 3 days after HSV-1 infection (Figure 4A). Histopathologic examination of the facial nerve displayed that mouse treated with WL-1 had a lower HSV-1-induced inflammation score, indicating that WL-1 reduced facial nerve injury (Figure 4B). Taken together, our results indicated that WL-1 can resist viral infection and thereby prevent the occurrence of facial paralysis.

## 4. Discussion

HSV-1 causes a wide range of infections from mild to life-threatening in the human population. There are effective treatments for HSV-1 infections, which are limited due to HSV-1 latency and the development of resistance to current therapeutics. It is urgent to develop new specific agents against HSV-1 infection due to the universality of HSV-1 infection and the limitations of existing clinical drugs (Sadowski et al., 2021). Antimicrobial peptides (AMPs) are widely distributed in many species and represent highly effective natural defenses that are central weapons for the host to resist infection (Nizet, 2006; Mookherjee and Hancock, 2007; Rossi et al., 2012). They have different mechanisms of action from traditional antibiotics, showing potent and broad-spectrum antibacterial, antifungal, and antiviral activities and are not easy to induce drug resistance (Neto et al., 2007; Aribi Al-Zoobaee et al., 2020). The cathelicidin family is one of the most important AMPs in the host immune defense system. So far, some of the cathelicidin-related drugs are undergoing clinical trials, indicating that cathelicidins have great potential to be developed as new antimicrobial drugs as alternatives to traditional antibiotics and medicines (Steinstraesser et al., 2011; Wang et al., 2019; Mookherjee et al., 2020; Díez-Aguilar et al., 2021; Bhattacharjya et al., 2022; Talapko et al., 2022). It has been reported that cathelicidins possess many important activities such as broad-spectrum and high antibacterial activity, anti-inflammation, and tissue damage inhibition (Auvynet and Rosenstein, 2009; Nijnik and Hancock, 2009; Wuerth and Hancock, 2011; Choi et al., 2012; Kahlenberg and Kaplan, 2013). In fact, a few cathelicidins have been found to have inhibitory effects on various viral infections, including hepatitis C virus, vaccinia virus, and human immunodeficiency virus 1. In this study, we revealed that WL-1, an antiviral peptide derived from cathelicidins with low toxicity, inhibits the HSV-1 infection *in vitro* and *in vivo*, adding antiviral activity to the broad-spectrum function of cathelicidins. Specifically, WL-1 can not only restrain viral replication but also prevent the pathological symptoms induced by HSV-1 infection. In addition, the anti-HSV-1 effect of WL-1 is superior to acyclovir, the most clinically used nucleoside analog against HSV-1 infection, at the same concentration. Our data have demonstrated that WL-1 may have great potential to be optimized as an anti-HSV-1 compound. Furthermore, it remains to be evaluated whether WL-1 has similar effects on other HSV-1 strains and DNA viruses.

Viral infection can be resisted in many ways by antimicrobial peptides, drugs, and other small-molecule inhibitors. For example, the antimicrobial peptide CATH-2 has been reported to modulate the inflammatory response by regulating the secretion of inflammatory cytokines and activation of the NLRP3 inflammasome, resulting in protection against IAV infection (Coorens et al., 2017; Peng et al., 2022). A defense peptide called An1a restricts dengue and Zika virus infection by inhibiting the viral NS2B-NS3 protease (Ji et al., 2019). Acyclovir inhibits the enzymatic activity of HSV thymidine kinase (TK), thereby interrupting viral DNA replication (Vashishtha and Kuchta, 2016; Sadowski et al., 2021). Moreover, Artemisia argyi leaf extract AEE destroys the membrane integrity of HSV-1 viral particles, resulting in impaired viral attachment and penetration (Liu et al., 2023). These results reveal various molecular mechanisms of host-defense peptides against viral infections, including direct viral killing, regulation of viral infection, and participation in host immune regulation against viral infections. We found that WL-1 treatment could reduce viral titer. So that we speculated that WL-1 may have the same anti-HSV-1 infection mechanism as LL-37, which has been described to damage the viral membrane envelope or inhibit HSV-1 adsorption to cells (Howell et al., 2004; Lee et al., 2014). Likewise, the administration of WL-1 obviously reduced the virus load and viral gene expression as well as the inflammatory factors production in cells and mice, which reminds us that WL-1 perhaps participates in the regulation of the viral replication cycle and inflammatory response caused by viral infection. In brief, the mechanism of WL-1 resistance against HSV-1 infection is more complicated and requires further research to discuss.

Today, multiple treatments and drugs have no significant effect on Bell's palsy, the most common form of peripheral facial palsy (Gronseth and Paduga, 2012; Zandian et al., 2014; Newadkar et al., 2016; Zhang et al., 2019). Antivirals and steroids were the most commonly prescribed medications in the early days of Bell's palsy (Jalali et al., 2021). There was no benefit from antiviral therapy alone compared with placebo (Gallo et al., 1997). When researchers compared the effect of steroids with placebo, they found that the reduction in the proportion of patients who did not fully recover at 6 months was small and not statistically significant (Gu et al., 2014). Combined oral steroids and antivirals were associated with a lower rate of incomplete recovery when compared with oral steroids alone (Gallo et al., 1997). Another study showed that patients treated with steroids combined with acyclovir had a higher overall recovery rate than those treated with steroids alone, but the difference was not statistically significant (Yeo et al., 2008). Because acyclovir's bioavailability is relatively low (15–30%), new drugs are being investigated (Newadkar et al., 2016). Surprisingly, we found that WL-1 can not only reduce the viral load of HSV-1 but also prevent the occurrence of facial palsy induced by infection, indicating that WL-1 can be used as a potential drug for treating Bell's palsy. Certainly, further studies are needed to evaluate the effect of WL-1 on Bell's palsy using other models.

## 5. Conclusion

In summary, the designed peptide WL-1 showed high antiviral abilities against HSV-1 infection and prevented the facial palsy occurrence induced by HSV-1 in mice. Therefore, it may be an excellent candidate or template for the development of a therapeutic agent to treat clinical infection of HSV-1. Much more effort should be made to understand the antiviral mechanism of WL-1.

## Data availability statement

The raw data supporting the conclusions of this article will be made available by the authors, without undue reservation.

## Ethics statement

The animal study was reviewed and approved by the Animal Care Committee of Shanxi Agricultural University, approval number: SXAU-EAW-2022M.RX.00906001.

## Author contributions

LW and XG: conceptualization and funding acquisition. XG: methodology and writing—original draft preparation. XG, YA, WT, LM, MW, JL, BL, and WH: investigation. LW: resources and writing—review and editing. All authors have read and agreed to the published version of the manuscript.
