# Corrigendum: Cathelicidin-derived antiviral peptide inhibits herpes simplex virus 1 infection

**DOI:** 10.3389/fmicb.2023.1254775

**Published:** 2023-07-25

**Authors:** Xiaomin Guo, Yanxing An, Wanmin Tan, Ling Ma, Mingyang Wang, Juyan Li, Binghong Li, Wei Hou, Li Wu

**Affiliations:** ^1^College of Veterinary Medicine, Shanxi Agricultural University, Jinzhong, China; ^2^Department of Medical Imaging, First Affiliated Hospital of Kunming Medical University, Kunming, China

**Keywords:** cathelicidins, antiviral peptide, HSV-1, facial palsy, WL-1

In the published article, there was a typographical error that stated that the dose of HSV-1 in ocular scarification mice was 5 × 10^4^ PFU, which should have been 4 × 10^4^ PFU.

A correction has been made to 2. Materials and methods, *2.7. Mice infection*, Paragraph 1. This sentence previously stated:

“The infection dose of HSV-1 was 5 × 10^4^ plaque forming units (PFU).”

The corrected sentence appears below:

“The infection dose of HSV-1 was 4 × 10^4^ plaque forming units (PFU).”

The authors apologize for this error and state that this does not change the scientific conclusions of the article in any way. The original article has been updated.

